# Molecular insights versus morphological traits: rethinking identification of the closely related *Angiostrongylus cantonensis* and *Angiostrongylus malaysiensis*

**DOI:** 10.1186/s13071-024-06140-9

**Published:** 2024-02-08

**Authors:** Chanisara Kaenkaew, Abigail Hui En Chan, Naowarat Saralamba, Jiraporn Ruangsittichai, Kittipong Chaisiri, Vachirapong Charoennitiwat, Urusa Thaenkham

**Affiliations:** 1 Department of Helminthology, Faculty of Tropical Medicine, Mahidol University, Bangkok, Thailand; 2 Department of Molecular Tropical Medicine and Molecular Genetics, Faculty of Tropical Medicine, Mahidol University, Bangkok, Thailand; 3 Department of Medical Entomology, Faculty of Tropical Medicine, Mahidol University, Bangkok, Thailand

**Keywords:** *Angiostrongylus cantonensis*, *Angiostrongylus malaysiensis*, Misidentification, Morphological and molecular characters, Hybrids

## Abstract

**Background:**

The closely related *Angiostrongylus cantonensis* and *Angiostrongylus malaysiensis* have been reported to coexist in Thailand and share similar hosts and life cycles. Recently, in an angiostrongyliasis outbreak in Thailand, both *A. cantonensis* and *A. malaysiensis* were found in the cerebrospinal fluid of affected patients. Morphological similarities, overlapping distribution, shared hosts and habitats, and the close genetics of the two *Angiostrongylus* species can complicate accurate species identification. Addressing these challenges, this study aims to evaluate whether a correlation between the morphological and genetic identities of *A. cantonensis* and *A. malaysiensis* can improve species identification accuracy.

**Methods:**

*Angiostrongylus* spp. specimens from five zoogeographical regions in Thailand were subjected to morphological and molecular identification using the mitochondrial cytochrome *b* gene and the nuclear internal transcribed spacer 2 region (ITS2). The morphological characters for males and females were then validated using the species identity obtained from the nuclear ITS2 region.

**Results:**

The results revealed that morphological misidentifications between these two closely related species are common due to overlapping morphological characters. Although certain male traits such as body length and width aided species differentiation, female traits were found to be less reliable. Furthermore, hybrid forms (8.2%) were revealed through the ITS2 results, which can further complicate morphological identification. Mito-nuclear discordance was also present in 1.9% of the *Angiostrongylus* specimens from Thailand, suggesting a complex historical interbreeding between the species.

**Conclusions:**

Based on our findings, we suggest that nuclear ITS2 is a reliable marker for species identification of *A. cantonensis* and *A. malaysiensis*, especially in regions where both species coexist. Additionally, the scope and consequences of hybridization between the two closely related *Angiostrongylus* species should be further investigated in Thailand.

**Graphical Abstract:**

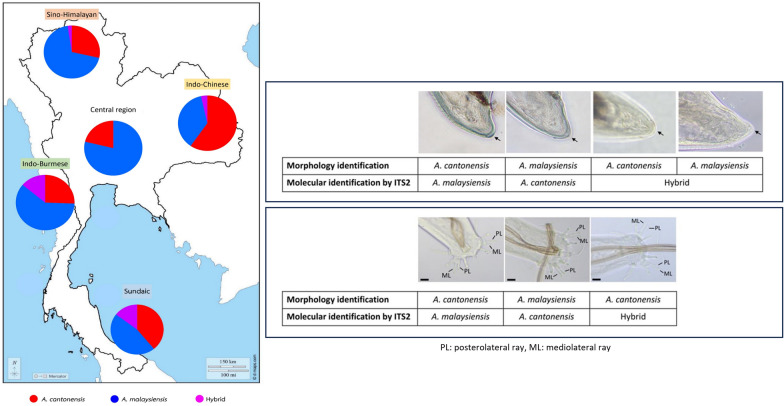

**Supplementary Information:**

The online version contains supplementary material available at 10.1186/s13071-024-06140-9.

## Background

*Angiostrongylus*, a nematode genus within the Metastrongyloidea superfamily and Angiostrongylidae family, hosts species that can pose substantial health risks to humans. Specifically, *Angiostrongylus cantonensis* and *Angiostrongylus costaricensis* can cause eosinophilic meningoencephalitis and abdominal angiostrongyliasis, respectively [1–3]. Their life cycles involve complex interactions with snail intermediate hosts and rodent definitive hosts, with humans as accidental hosts after consuming infected snails [1]. *Angiostrongylus malaysiensis*, a closely related species of *A. cantonensis*, has been proposed as a potential human pathogen. However, additional research is necessary to ascertain the pathogenicity of *A. malaysiensis* [4, 5].

In Thailand, three *Angiostrongylus* species have been reported, namely *A. cantonensis*, *A. malaysiensis*, and *A. siamensis.* Accurate identification is essential, as misidentification can lead to improper diagnosis and treatment, thereby exacerbating public health issues [6, 7]. The morphological similarities and the overlapping habitats of *A. cantonensis* and *A. malaysiensis* have complicated the accurate identification of these species. Species identification becomes even more challenging with the discovery of both *A. cantonensis* and *A. malaysiensis* within a single snail host [4, 5]. Notably, in a recent angiostrongyliasis outbreak in Kalasin Province, Thailand, both *A. cantonensis* and *A. malaysiensis* were found in the cerebrospinal fluid of affected patients [5].

Previously, morphological traits served as the gold standard for *Angiostrongylus* species identification. However, as mentioned earlier, challenges in accurate identification can arise, leading to misidentification even with careful morphological examination. Consequently, molecular methods are necessary for confirmation [8–11]. Additionally, reports of F1 hybridization between *A. cantonensis* and *A. malaysiensis* highlight the potential for morphological uncertainty [12]. To address the issue of misidentification, molecular identification using reliable genetic markers is essential. Promising markers include the nuclear ribosomal internal transcribed spacer 1 (ITS1) region, 18S ribosomal RNA (rRNA) gene, and mitochondrial genes such as the cytochrome *c* oxidase subunit 1 (*COI*), cytochrome *b* (*cytb*), and 16S rRNA genes [6, 9, 13–16]. Recently, a SYBR Green-based quantitative polymerase chain reaction (qPCR) method utilizing species-specific primers targeting the mitochondrial *cytb* gene was developed to quantitatively detect and differentiate between *A. cantonensis* and *A. malaysiensis* [4].

In response to these challenges, our study aims to determine whether a correlation between the morphological and genetic identities of *A. cantonensis* and *A. malaysiensis* can improve species identification accuracy. By comparing morphological diagnostic characters with their corresponding genetic identity, we hypothesize that validated morphological diagnostic characters directly associated with their molecular identity can aid in accurate species identification. To test this hypothesis, adult worms from five distinct zoogeographical regions in Thailand were collected and initially identified based on morphological traits. Molecular methods subsequently confirmed the species’ identity using the mitochondrial *cytb* gene and the nuclear internal transcribed spacer 2 (ITS2) region. By integrating morphological and molecular data, we hope to provide valuable insight into improving species identification, offering a more comprehensive and reliable approach to overcoming the challenges currently associated with *Angiostrongylus* identification.

## Methods

### Study area and specimens used

This study was conducted using 257 archived specimens of *Angiostrongylus* adult worms. These specimens, collected from five distinct zoogeographical regions in Thailand, were initially acquired as part of a previous study (no. FTM-ACUC 002/2013). An overview of the specimens categorized by region is provided in Table 1. For each region, a minimum of 10 specimens were selected for analysis. In instances where fewer than 10 specimens were available, all existing samples were included in the study.

**Table 1:** *Angiostrongylus* spp. specimens used for morphological and molecular identification

Zoogeographical region	Province^a^	Male	Female
Sino-Himalayan (*n* = 42)	Chiang Mai (CMI)	4	0
	Nan (NAN)	10	15
	Phayao (PYO)	3	10
Indo-Burmese (*n* = 43)	Prachuap Khiri Khan (PKN)	4	0
	Kanchanaburi (KRI)	9	0
	Ratchaburi (RBR)	10	20
Indo-Chinese (*n* = 57)	Nong Khai (NKI)	1	0
	Khon Kaen (KKN)	10	2
	Chanthaburi (CTI)	10	0
	Nakhon Phanom (NPM)	10	0
	Maha Sarakham (MKM)	20	4
Sundaic (*n* = 82)	Phang-Nga (PHA)	1	13
	Nakhon Si Thammarat (NKT)	10	36
	Ranong (RNG)	10	0
	Narathiwat (NWT)	5	0
	Satun (STN)	7	0
Central (*n* = 33)	Lopburi (LRI)	5	0
	Bangkok (BKK)	0	7
	Phitsanulok (PLK)	21	0
Total (*n* = 257)		150	107

^a^Abbreviations for each province are in parentheses

### Morphological identification

Archived *Angiostrongylus* adult worms previously preserved in 80% ethanol were morphologically identified following a checklist and diagnostic characters detailed in the morphometry of pulmonary angiostrongylids in Southeast Asia [17–20]. For males, the specific diagnostic characters used for identification were spicule length and Δ bursal rays (length difference between the posterolateral (PL) ray and the mediolateral (ML) ray). For females, three specific diagnostic characters were used: the distance of the anus opening from the posterior end, the distance of the vulva opening from the posterior end, and the presence of a minute projection at the posterior end. In addition, four morphological characters were measured for both sexes: body length, body width, esophagus length, and esophagus width. Measurements were carried out using a stereomicroscope (Olympus SZ51) and an inverted compound microscope (ZEISS Primovert).

### Molecular identification

#### DNA extraction

To isolate genomic DNA (gDNA), individual adult *Angiostrongylus* were thoroughly rinsed with sterile distilled water to eliminate any ethanol residue and placed in a 1.7-ml centrifuge tube. DNA extraction was then carried out using the Genomic DNA Mini Kit (Geneaid Biotech Ltd., Taipei, Taiwan), adhering to the manufacturer’s guidelines. The gDNA was stored at 4 °C until further analysis.

#### SYBR Green quantitative real-time PCR using the *cytb* gene

Molecular identification of *A. cantonensis* and *A. malaysiensis* was determined using the mitochondrial *cytb* gene following the species-specific primers (AC4_cytb_F: 5′- AATGTTTGTTGAGGCAGATC-3′ and AC5_cytb_R: 5′-GCTACAACACCCATAACCT-3′ for *A. cantonensis*; AM3_cytb_F: 5′-CGAGATATTTATTGAGGCTG-3′ and AM4_cytb_R: 5′-GACAAAACCCTCATCAATAA-3′ for *A. malaysiensis*) and the SYBR Green qPCR assay developed by Jakkul et al. [4]. All gDNA samples were diluted to 1 ng and used as the DNA template for qPCR. The qPCR reactions for both species-specific primers were conducted concurrently on a CFX96 Touch™ Real-Time PCR system (Bio-Rad Laboratories, CA, USA).

#### PCR–RFLP using the ITS2 region

Molecular identification of *A. cantonensis* and *A. malaysiensis* with the ITS2 region of the nuclear ribosomal gene was carried out via the PCR restriction fragment length polymorphism (PCR–RFLP) method. Primers (AngiITS2_forward: 5′-ACATCTGGTTCAGGGTTGTT-3′ and AngiITS2_reverse: 5′-AGCATACAAGCACATGATCAC-3′) and thermal cycling conditions were applied following Rodpai et al. (2016). The PCR reactions were performed on a T100™ Thermal Cycler (Bio-Rad Laboratories, CA, USA). Once the 395-bp PCR product was obtained, the PCR–RFLP method using the *Bts*I-v2 restriction enzyme (New England Biolabs, MA, USA) was then used for species differentiation between *A. cantonensis* and *A. malaysiensis*. Potential hybridization between *A. cantonensis* and *A. malaysiensis* was also investigated by comparing the specific band patterns attributed to each species. The restriction enzyme cutting sites are illustrated in Additional file 1: Fig. S1. The 30 μl restriction digest mixture contained one unit of *Bts*I-v2 restriction enzyme (New England Biolabs, MA, USA), 1× CutSmart^®^ Buffer (New England Biolabs, MA, USA), 100 ng/μl of the purified PCR product, and nuclease-free water. The resulting PCR–RFLP band patterns were visualized on a 2% agarose gel stained with SYBR™ Safe (Thermo Fisher Scientific, MA, USA).

Additionally, sequencing of potential hybrids were performed by a commercial company (Tsingke Biotech, Beijing, China) using the FastNGS method to confirm the presence of both *A. cantonensis* and *A. malaysiensis* DNA and to detect the presence of double peaks in the electropherogram.

### Statistical analysis

Following molecular identification, a cross-verification was initiated to confirm the morphological identity of the specimens. The cross-verification and validation of morphological diagnostic characters were performed using three molecularly defined groups obtained from the nuclear ITS2 results: *A. cantonensis*, *A. malaysiensis*, and their hybrid. All data derived from morphological identification, except for the presence or absence of minute projections at the posterior end of the female, were utilized to validate the diagnostic characters.

For each diagnostic character, a boxplot was generated to compare the morphological differences among the three molecularly defined groups. The normality of data was assessed, and differences among the groups based on various morphological diagnostic characters were analyzed using analysis of variance (ANOVA) with Bonferroni’s post hoc analysis. The results are presented as mean and standard deviation (SD) values, with a predetermined significance level set at *P* < 0.05. Principal component analysis (PCA) was conducted for the males and females using the morphological measurements obtained to determine whether the three molecularly defined groups could be differentiated based on morphology. The morphological measurements used for PCA for males included body length, body width, esophagus length, esophagus width, spicule length, and Δ bursal rays. The morphological characters used for females were body length, body width, esophagus length, esophagus width, distance of the anus opening from the posterior end, and distance of the vulva opening from posterior end. All statistical analysis and PCA were conducted using R Studio v.1.2.5033 [21].

## Results

### Morphological identification of *Angiostrongylus*

In total, 257 adult worms, comprising 150 males and 107 females, sourced from five different zoogeographical regions, were examined for morphological identification. From the results, it was discerned that 35.8% (92/257) of the specimens were identified as *A. cantonensis*, while 64.2% (165/257) were identified as *A. malaysiensis* (Table 2 and Additional file 2: Table S1).

**Table 2:** Morphological and molecular identification of *Angiostrongylus* species

Region	No.	Morphological identification		Molecular identification					Misidentification by morphological characters (%)
				*cytb* gene (mtDNA)		ITS2 (nuDNA)			
		*Ac* (%)	*Am* (%)	*Ac* (%)	*Am* (%)	*Ac* (%)	*Am* (%)	Hybrid (%)	
Sino-Himalayan	42	26.2	73.8	23.8	76.2	28.6	69.0	2.4	33.3
Indo-Burmese	43	32.6	67.4	18.6	81.4	25.6	60.5	13.9	32.5
Indo-Chinese	57	36.8	63.2	59.6	40.4	59.6	36.9	3.5	42.1
Sundaic	82	39.0	61.0	39.0	61.0	39.0	46.3	14.7	37.8
Central	33	42.4	57.6	78.8	21.2	78.8	21.2	0	36.3
Total	257	35.8	64.2	42.8	57.2	44.7	47.1	8.2	36.9

*Angiostrongylus cantonensis* and *A. malaysiensis* are abbreviated as *Ac* and *Am*, respectively

*mtDNA* mitochondrial DNA, *nuDNA* nuclear DNA

### Molecular identification of *Angiostrongylus* using the *cytb* gene and ITS2 region

Molecular analysis using qPCR targeting the *cytb* gene revealed that 42.8% (110/257) of the specimens were identified as *A. cantonensis*, while 57.2% (147/257) were identified as *A. malaysiensis*. The PCR–RFLP analysis of the ITS2 amplicons indicated that 44.7% (115/257) of the worms were *A. cantonensis*, 47.1% (121/257) were *A. malaysiensis*, and 8.2% (21/257) represented hybrids of *A. cantonensis* and *A. malaysiensis* (Table 2 and Additional file 2: Table S1). A comparison of species identity between the two genetic markers revealed that 89.8% (231/257) exhibited either pure *A. cantonensis* or pure *A. malaysiensis*, as indicated by a congruence of species identity using both the *cytb* gene and ITS2 region. In contrast, discordant hybridization was noted in 1.9% (5/257) of the specimens, as characterized by discrepancies in the molecular identification results between the *cytb* gene and the ITS2 region. The band patterns indicative of congruence between the two genetic markers and discordant hybridization, along with potential F1 hybrids, are depicted in Additional file 3: Fig. S2.

**Fig. 1:**
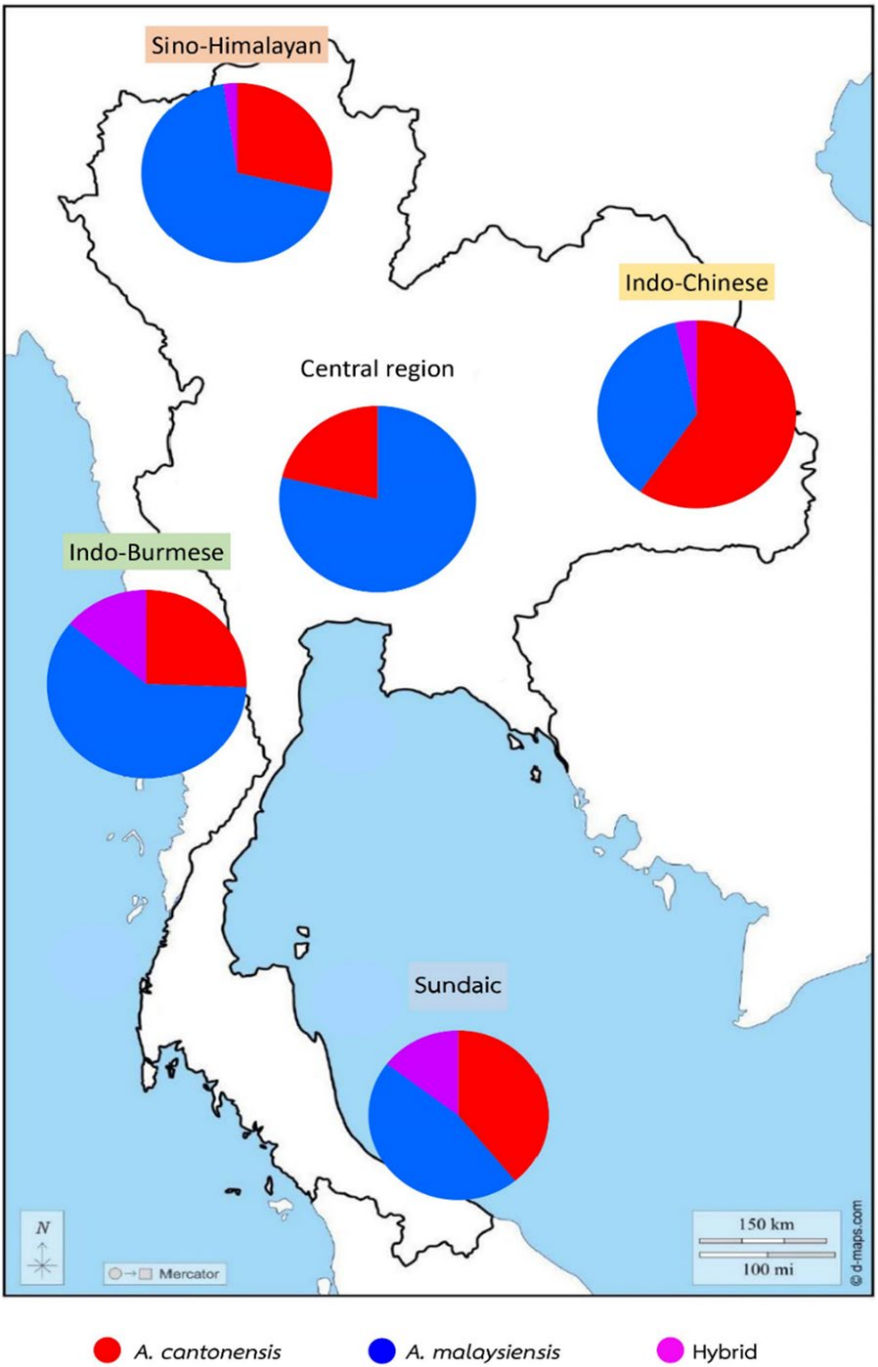
The distribution of *A. cantonensis*, *A. malaysiensis*, and their hybrid across the zoogeographical regions using ITS2 as a genetic marker for molecular identification

After molecular identification, it was found that 36.9% (95/257) of the specimens were misidentified based on morphological characteristics. Among those misidentified, the majority (52.6%, 50/257) were initially morphologically identified as *A. malaysiensis* but were subsequently molecularly identified as *A. cantonensis*. The misidentification errors were more common for male adults relative to females. Furthermore, within the specimens identified as hybrids based on the ITS2 region, a significant proportion (18.9%, 18/95) were initially identified morphologically as *A. malaysiensis*. Among these misidentified hybrids, 11 were males and seven were females. Based on ITS2 as the genetic marker, the numbers of *A. cantonensis* and *A. malaysiensis* tended to be approximately equal, as depicted in Fig. 1.

### Confirmation of potential hybrids of *A. cantonensis* and *A. malaysiensis*

**Fig. 2:**
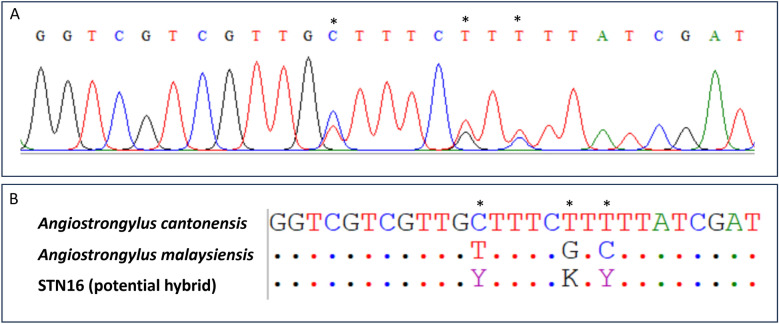
Evidence of the hybrid form of *A. cantonensis* and *A. malaysiensis* in the **A** electropherogram and **B** sequence alignment. An asterisk (*) indicates the position of the double peaks observed at the fixed difference positions

DNA sequencing of the ITS2 amplicons of potential hybrids revealed the presence of double peaks in the electropherograms obtained, confirming the hybrid status of the specimens in Thailand (Fig. 2A). The double peaks were obtained at three fixed difference positions between the ITS2 sequences of *A. cantonensis* and *A. malaysiensis* (Fig. 2B). For *A. cantonensis*, the nucleotides at the fixed difference positions were C, T, and T, while for *A. malaysiensis*, the nucleotides were T, G, and C. The potential hybrid specimen (STN16) showed double peaks of both nucleotides from *A. cantonensis* and *A. malaysiensis* (C/T, T/G, and T/C), thereby confirming its hybrid nature.

### Validation of the morphological diagnostic characteristics after genetic status confirmation

**Fig. 3:**
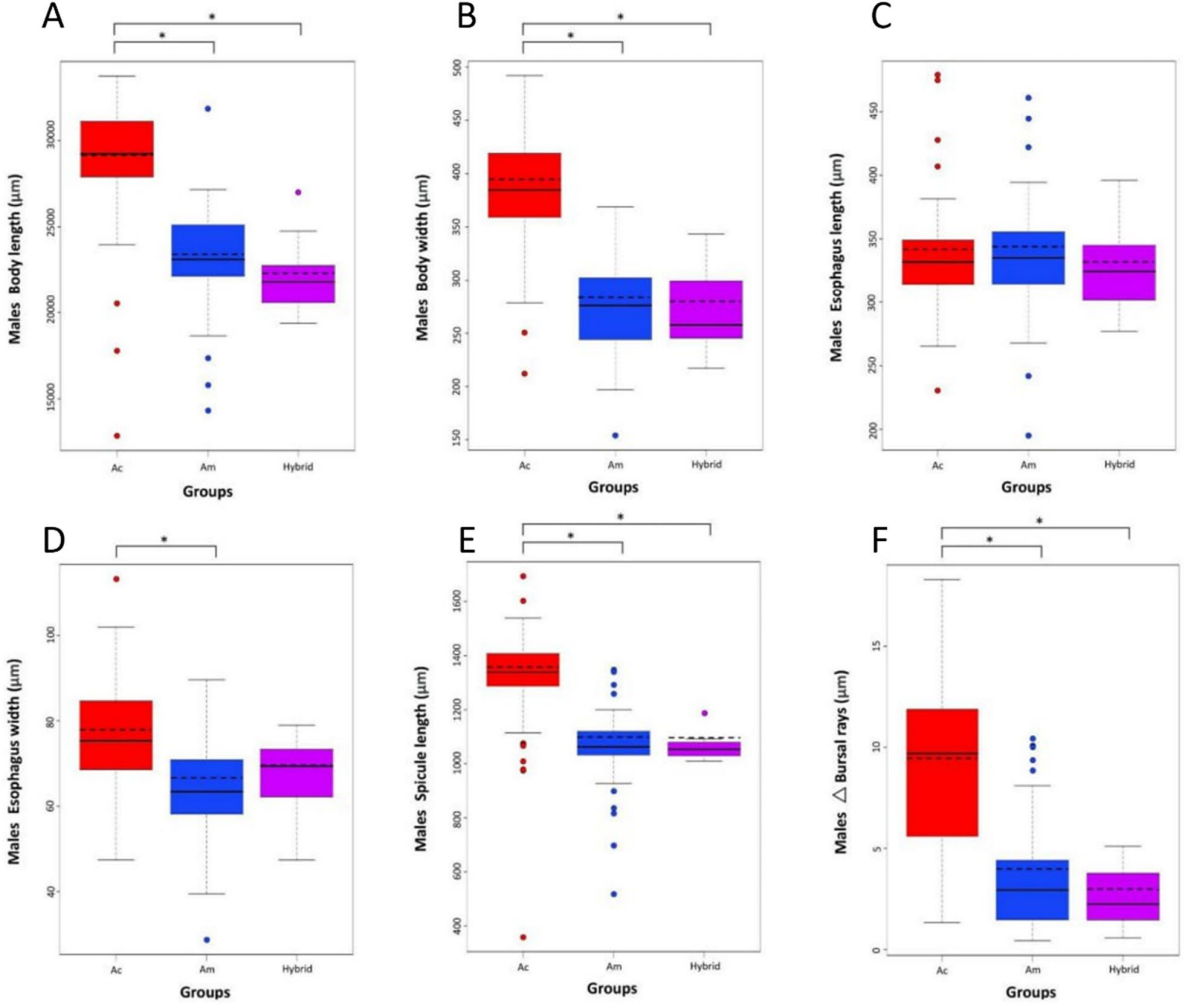
Comparative analysis of six male morphological characters. **A** body length, **B** body width, **C** esophagus length, **D** esophagus width, **E** spicule length, and **F** △ bursa rays according to molecular identity with the nuclear ITS2 region. An asterisk (*) indicates a statistical significance of *P* < 0.05 between the means of each group. The solid line of the boxplot indicates the mean value obtained from the morphological measurements, while the dashed line indicates the median. *A. cantonensis* and *A. malaysiensis* are abbreviated as Ac and Am, respectively

Upon confirmation of the molecular identity based on the ITS2 region, comparisons with six morphological diagnostic characters revealed that the male specimens were statistically significantly different (*P* < 0.05) in body length, body width, spicule length, and bursal rays across all groups, which included *A. cantonensis, A. malaysiensis*, and their hybrid. For esophagus width, there was a significant difference between *A. cantonensis* and *A. malaysiensis.* In contrast, the esophagus length did not vary significantly across all groups. Notably, the use of spicule length as a diagnostic character resulted in numerous outliers, which can confound identification. Moreover, substantial overlap in measurements between *A. cantonensis* and *A. malaysiensis* was observed for △ bursa rays (Fig. 3), which is a key diagnostic character for morphological differentiation between the two species. The morphometric comparison values among the three groups are presented in Additional file 4: Table S2.

PCA using six male morphological characters revealed that *A. cantonensis* and *A. malaysiensis* can be differentiated, but an overlapping region could confound identification (Additional file 5: Fig. S3). Moreover, the potential hybrids could not be differentiated from *A. malaysiensis*, as the PCA graph revealed that the hybrid specimens fell within the range of *A. malaysiensis*.

**Fig. 4:**
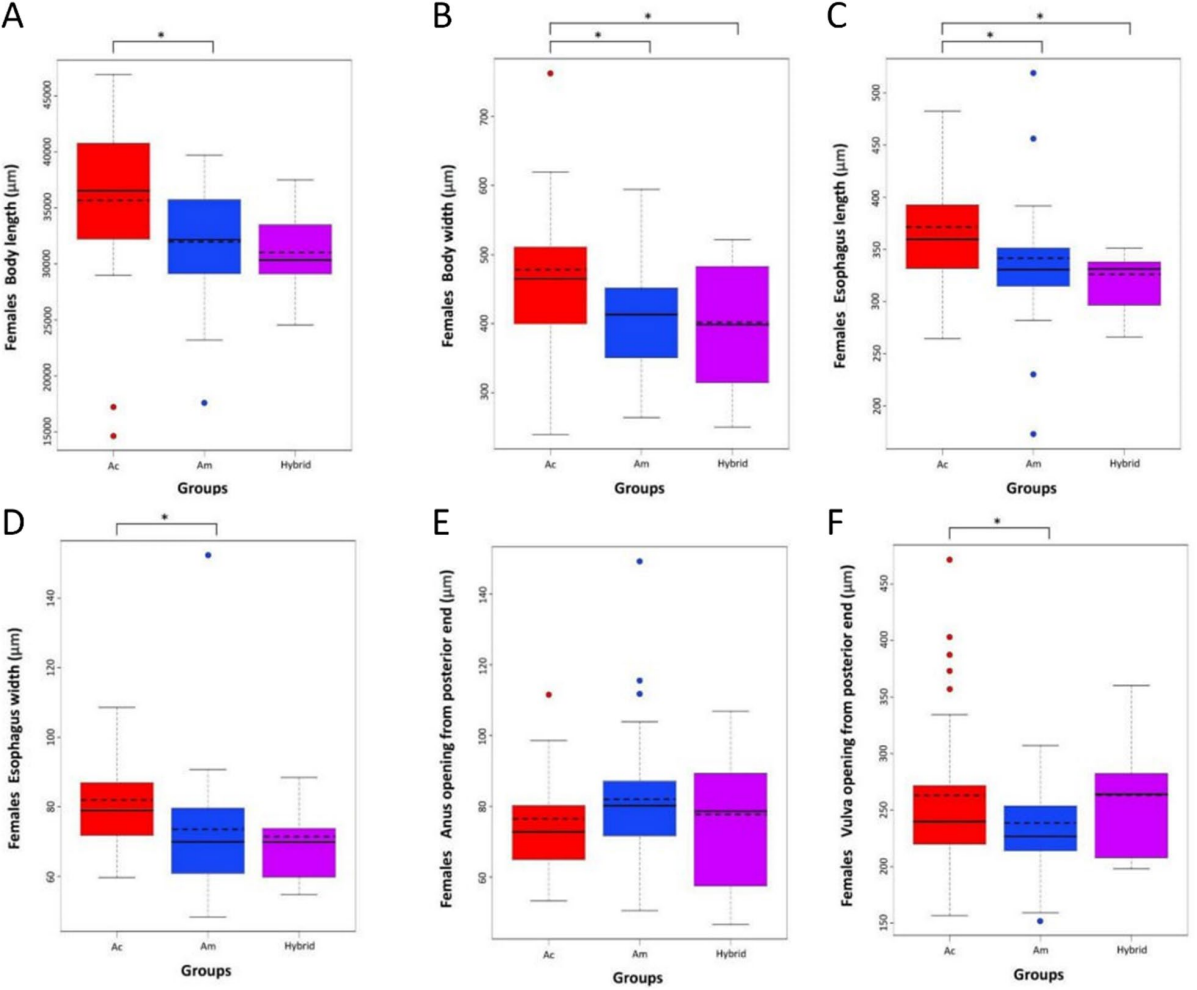
Comparative analysis of six female morphological characters: **A** body length, **B** body width, **C** esophagus length, **D** esophagus width, **E** distance from the anus to the posterior end, and **F** distance from the vulva to the posterior end according to molecular identity with the nuclear ITS2 region. An asterisk (*) indicates a statistical significance of *P* < 0.05 between the means of each group. The solid line of the boxplot indicates the mean value obtained from the morphological measurements, while the dashed line indicates the median. *Angiostrongylus cantonensis* and *A. malaysiensis* are abbreviated as Ac and Am, respectively

For females, statistically significant differences (*P* < 0.05) in body width and esophagus length were observed across the three groups. Significant differences were found between *A. cantonensis* and *A. malaysiensis* in body length, esophagus width, and distance from the vulva opening to the posterior end. Despite showing statistically significant differences using the morphological characters mentioned above, there was no distinct differentiation between the *A. cantonensis, A. malaysiensis*, and hybrid groups due to the overlap in the range of measurements obtained (Fig. 4). The morphometric comparison values among the three groups are shown in Additional file 4. PCA of the six female morphological characters provided further evidence that *A. cantonensis*, *A. malaysiensis*, and their hybrids could not be differentiated (Additional file 6: Fig. S4).

**Fig. 5:**
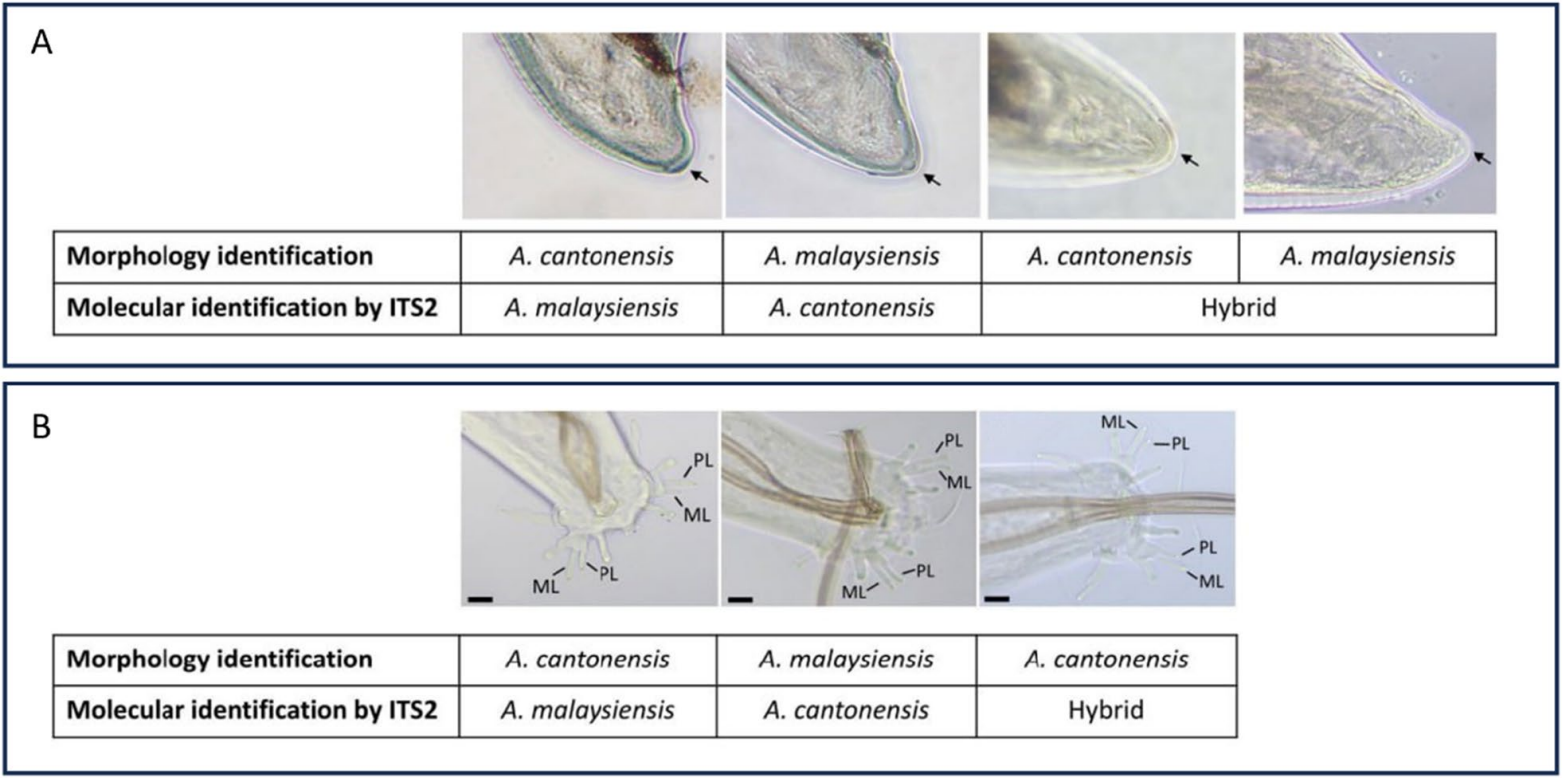
Disparities between morphological traits and molecular identification of *Angiostrongylus* spp. **A** males and **B** females. Actual species identity was determined by molecular identification using the nuclear ITS2 region. The scale bars indicate a length of 20 µm; the arrowheads point to the tips of the female posterior ends, signifying the absence or presence of minute projections, while the posterolateral (PL) and mediolateral (ML) rays are labeled for males

To highlight instances of initial morphological misidentification, Fig. 5 presents the disparities between the morphological traits and molecular identification for both males and females. For females, the specimens shown were initially morphologically identified due to the absence or presence of a minute projection at the tip of the posterior end, but subsequent molecular identification revealed that the morphological identity did not correspond to the molecular identity (Fig. 5A). Likewise, in males, the specimens in Fig. 5B were initially identified as either *A. cantonensis* or *A. malaysiensis* due to observed disparities in the length of the PL and ML rays. However, molecular analysis confirmed that the morphological characters were not reliable and did not match the molecular identity.

## Discussion

In this study, morphological differentiation between *A. cantonensis* and *A. malaysiensis* was challenging. Morphological misidentifications were particularly frequent for *A. malaysiensis* after confirmation of species identity using the nuclear ITS2 region. Despite thorough efforts, the misidentification rate was 36.9% across the specimens studied. In males, the key morphological character typically used to differentiate *A. cantonensis* and *A. malaysiensis* is the difference in the lengths between the PL and ML rays (△ bursa rays). However, our results revealed that this key morphological character may not be sufficient for species differentiation. A similar phenomenon was observed in females, where the unique minute projection, a prominent morphological character of *A. malaysiensis*, failed to distinguish it from *A. cantonensis*. Of the morphological characters, our findings revealed that the male body length and width can effectively differentiate between the two species. Contrarily, for females, size overlap between the two species was observed, posing challenges for effective species differentiation.

The use of morphological characters for species identification between the two closely related *A. cantonensis* and *A. malaysiensis* is further complicated by the presence of hybrid forms of *A. cantonensis* and *A. malaysiensis* in Thailand. Of the representative specimens, 8.2% were identified as hybrids of *A. cantonensis* and *A. malaysiensis* by the nuclear ITS2 region. The overlapping distribution of *A. cantonensis* and *A. malaysiensis* in Indo-China has been highlighted in past studies, with F1 hybrids found in natural populations in Thailand [12, 22]. Moreover, co-infection of the two species have been discovered in both aquatic and terrestrial snails, with a report of the co-occurrence of both *A. cantonensis* and *A. malaysiensis* DNA from the cerebrospinal fluid of patients [4, 5]. Our results show that hybrids mainly displayed morphological characteristics resembling *A. malaysiensis*, which can confound species identification. Moreover, overlaps in morphological characters among *A. cantonensis*, *A. malaysiensis*, and their hybrids were observed. Evidence of hybridization has been discovered in other parasitic nematodes as well, such as *Haemonchus contortus* and *Haemonchus placei*, and in the sibling species of *Anisakis simplex* and *Anisakis pegreffi* [23, 24].

Aside from the ineffectiveness of morphological characters for species differentiation between *A. cantonensis*, *A. malaysiensis*, and their hybrid forms, mito-nuclear discordances were observed based on molecular identification using the nuclear ITS2 region and the mitochondrial *cytb* gene. Mito-nuclear discordance is defined as conflicting results and differences in patterns of differentiation obtained from mitochondrial and nuclear DNA [25, 26]. Evidence of mito-nuclear discordance in *A. cantonensis* and *A. malaysiensis* in Thailand suggests that a complex relationship between these two closely related species is in play, possibly due to the sharing of hosts, habitats, geographical distribution, and their close genetics. Additionally, the evidence of introgressive hybridization, especially the observed discordance, can hint at complex interbreeding events and history between the two species in Thailand. Although our results revealed that the majority (89.8%) of the specimens were congruent between the species identity obtained from the mitochondrial and nuclear DNA markers, these findings, aside from pure ancestry lineages, are also suggestive of potential concordant hybridization events.

The use of nuclear DNA instead of mitochondrial DNA is proposed herein as a reliable method for species identification, especially in regions where closely related species such as *A. cantonensis* and *A. malaysiensis* coexist. The emphasis on nuclear DNA is pivotal, as it reveals potential hybridization events, which are expected to occur frequently. Moreover, misidentification due to hybridization discordance could occur if species identification relied solely on the mitochondrial gene [27, 28]. Regarding the distribution of *A. cantonensis* and *A. malaysiensis* in Thailand, our results revealed that the proportion of each species is approximately equal. Consequently, the previous understanding of *A. cantonensis* as the dominant species in this region may need reconsideration [22]. Additionally, greater scrutiny and further investigation are deemed necessary to accurately identify *Angiostrongylus* species in Thailand and neighboring regions. The study thus recommends using nuclear DNA over mitochondrial genes for accurate species identification of *A. cantonensis* and *A. malaysiensis*, especially in regions with overlapping cryptic species.

## Conclusions

In conclusion, differentiating *A. cantonensis* from *A. malaysiensis* based on morphology led to misidentifications. While certain male traits such as body length and width aided species differentiation, female traits were less reliable. Moreover, the discordance between nuclear and mitochondrial DNA indicated historical interbreeding between the species. We recommend using nuclear DNA over mitochondrial DNA for species identification of *A. cantonensis* and *A. malaysiensis* to reduce misidentification due to hybrid forms. Future research should focus on refining markers for differentiation, widening the scope and effects of hybridization, and investigating the ecological and health repercussions of hybridization between the two closely related species.

### Supplementary Information

**Additional file 1: Figure S1.** Illustration of the ITS2 PCR–RFLP band patterns for *A. cantonensis*, *A. malaysiensis*, and their hybrid form

**Additional file 2: Table S1** Morphological and molecular identification of *Angiostrongylus* spp. specimens

**Additional file 3: Figure S2.** Examples of ITS2 PCR–RFLP band patterns for specimens showing potential hybrid forms, congruence between the genetic markers, and discordant hybridization

**Additional file 4: Table S2.** Morphometric comparisons of *Angiostrongylus* spp. after species identity validation using the nuclear ITS2 region

**Additional file 5: Figure S3.** PCA of male morphological traits for *A. cantonensis*, *A, malaysiensis*, and their hybrid form

**Additional file 6: Figure S4.** PCA of female morphological traits for *A. cantonensis*, *A. malaysiensis*, and their hybrid form

## Data Availability

All data generated in this study are included in this published article and its supplementary information files.

## Abbreviations

*ANOVA*: Analysis of variance
*COI*: Cytochrome *c* oxidase subunit 1
*cytb*: Cytochrome *b*
ITS1: Internal transcribed spacer 1
ITS2: Internal transcribed spacer 2
ML: Mediolateral
PCA: Principal component analysis
PCR–RFLP: Polymerase chain reaction–restriction fragment length polymorphism
PL: Posterolateral
qPCR: Quantitative PCR
SD: Standard deviation

## References

[1] Barratt J, Chan D, Sandaradura I, Malik R, Spielman D, Lee R (2016). *Angiostrongylus cantonensis*: a review of its distribution, molecular biology and clinical significance as a human pathogen. Parasitology.

[2] da Silva AJ, Morassutti AL (2021). *Angiostrongylus* spp. (Nematoda: Metastrongyloidea) of global public health importance. Res Vet Sci..

[3] Rojas A, Maldonado-Junior A, Mora J, Morassutti A, Rodriguez R, Solano-Barquero A (2021). Abdominal angiostrongyliasis in the Americas: fifty years since the discovery of a new metastrongylid species, *Angiostrongylus*
*costaricensis*. Parasit Vectors.

[4] Jakkul W, Chaisiri K, Saralamba N, Limpanont Y, Dusitsittipon S, Charoennitiwat V (2021). Newly developed SYBR Green-based quantitative real-time PCRs revealed coinfection evidence of *Angiostrongylus*
*cantonensis* and *A*. *malaysiensis* in *Achantina*
*fulica* existing in Bangkok Metropolitan, Thailand. Food Waterborne Parastol..

[5] Watthanakulpanich D, Jakkul W, Chanapromma C, Ketboonlue T, Dekumyoy P, Lv Z (2021). Co-occurrence of *Angiostrongylus malaysiensis* and *Angiostrongylus cantonensis* DNA in cerebrospinal fluid: Evidence from human eosinophilic meningitis after ingestion of raw snail dish in Thailnad. Food Waterborne Parasitol.

[6] Chan AHE, Chaisiri K, Dusitsittipon S, Jakkul W, Charoennitiwat V, Komalamisra C (2020). Mitochondrial ribosomal genes as novel genetic markers for discrimination of closely related species in the *Angiostrongylus cantonensis* lineage. Acta Trop.

[7] Panadian D, Najer T, Modrý D (2023). An overview of *Angiostrongylus cantonensis* (Nematoda: Angiostrongylidae), an emerging cause of human angiostrongylosis on the Indian subcontinent. Pathogens.

[8] Aghazadeh M, Traub RJ, Mohandas N, Aland KV, Reid SA, McCarthy JS (2015). The mitochondrial genome of *Angiostrongylus mackerrasae* as a basis for molecular, epidemiological and population genetic studies. Parasit Vectors.

[9] Yong HS, Song SL, Eamsobhana P, Lim PE (2016). Complete mitochondrial genome of *Angiostrongylus malaysiensis* lungworm and molecular phylogeny of Metastrongyloid nematodes. Acta Trop.

[10] Dusitsittipon S, Criscion CD, Morand S, Komalamisra C, Thaenkham U (2018). Hurdles in the evolutionary epidemiology of *Angiostrongylus cantonensis*: Pseudogenes, incongrence between taxonomy and DNA sequence variants, and cryptic lineages. Evol Appl.

[11] Valentyne H, Spratt DM, Aghazadeh M, Jones MK, Slapeta J (2020). The mitochondrial genome of *Angiostrongylus mackerrasae* is distinct from *A. cantonensis* and *A. malaysiensis*. Parasitology..

[12] Dusitsittipon S, Criscion CD, Morand S, Komalamisra C, Thaenkham U (2017). Cryptic lineage diversity in the zoonotic pathogen *Angiostrongylus cantonensis*. Mol Phylogenet Evol.

[13] Qvarnstom Y, Sullivan J, Bishop H, Hollingsworth R, da Silva A (2007). PCR-based detection of *Angiostrongylus cantonensis* in tissue and mucus secretions from molluscan hosts. Appl Environ Microbiol.

[14] Eamsobhana P, Lim PE, Solano G, Zhang H, Gan X, Yong HS (2010). Molecular differentiation of *Angiostrongylus* taxa (Nematoda: Angiostrongylidae) by cytochrome c oxidase subunit I (*COI*) gene sequences. Acta Trop.

[15] Dusitsittipon S, Thaenkham U, Watthanakulpanich D, Adisakwattana P, Kamalamisra C (2015). Genetic differences in the rat lungworm, *Angiostrongylus cantonensis* (Nematoda: Angiostrongylidae) in Thailand. J Helminthol.

[16] Apichat V, Narongrit S, Jittranuch T, Anucha W, Wilaiwan P, Chamaiporn F (2016). Phylogeny of *Angiostrongylus cantonensis* in Thailand based on cytochrome c oxidase subunit I gene sequence. Southeast Asian J Trop Med Public Health.

[17] Chen HT (1935). Un Noveau nematode pulmonaire, *Pulmonema cantonensis* n.g., n. sp. des rats de Canton. Ann Parasitol Hum Comp..

[18] Bhaibulaya M, Cross JH (1971). *Angiostrongylus malaysiensis* (Nematoda: Metastrongylidae), a new species of rat lung-worm from Malaysia. Southeast Asian J Trop Med Public Health.

[19] Bhaibulaya M, Cross J (1979). Morphology and taxonomy of major *Angiostrongylus* species of eastern Asia and Australia. Studies on Angiostrongyliasis in Eastern Asia and Australia.

[20] Gibbons LM, Krishanasamy M (1986). *Malayometastrongylus diardinematus* n.g., n. sp. (Metastrongyloidea: Angiostrongylidae) from *Rattus rattus diardii* in Malaysia and a redescription of *Thaistrongylus harinasutai* Ohbayashi, Kamiya & Bhaibulaya, 1979. Syst Parasitol.

[21] RStudio Team. (2020). RStudio: integrated development for R.

[22] Rodpai R, Intapan PM, Thanchomnang T, Sanpool O, Sadaow L, Laymaniyong S (2016). *Angiostrongylus*
*cantonensis* and *A*. *malaysiensis* broadly overlap in Thailand, Lao PDR, Cambodia and Myanmar: a molecular survey of larvae in land snails. PLoS ONE..

[23] Chaudhry U, Redman EM, Abbas M, Muthusamy R, Ashraf K, Gilleard JS (2015). Genetic evidence for hybridization between *Haemonchus contortus* and *Haemonchus placei* in natural field populations and its implications for interspecies transmission of anthelmintic resistance. Int J Parasitol.

[24] Roca-Geronès X, Alcover MM, Godínez-González C, Montoliu I, Fisa R (2021). Hybrid genotype of *Anisakis*
*simplex* (s.s.) and *A*. *pegreffii* identified in third- and fourth-stage larvae from sympatric and allopatric Spanish marine waters. Animals (Basel)..

[25] Toews DPL, Brelsford A (2012). The biogeography of mitochondrial and nuclear discordance in animals. Mol Ecol.

[26] Després L (2019). One, two or more species? Mitonuclear discordance and species delimitation. Mol Ecol.

[27] Shults P, Hopken M, Eyer P, Blumenfeld A, Mateos M, Cohnstaedt LW (2022). Species delimintation and mitonuclear discordance within a species complex of biting midges. Sci Rep.

[28] Abalde S, Crocetta F, Tenorio MJ, D’Aniello S, Fassio G, Rodríguez-Flores PC (2023). Hidden species diversity and mito-nuclear discordance within the Mediterranean cone snail, *Lautoconus*
*ventricosus*. Mol Phy Evol.

