# Supplementary figures and images for: Molecular insights versus morphological traits: rethinking identification of the closely related Angiostrongylus cantonensis and Angiostrongylus malaysiensis
Source: Parasit Vectors. 2024 Feb 8;17:56. doi: 10.1186/s13071-024-06140-9 (PMC10851580; doi:10.1186/s13071-024-06140-9)

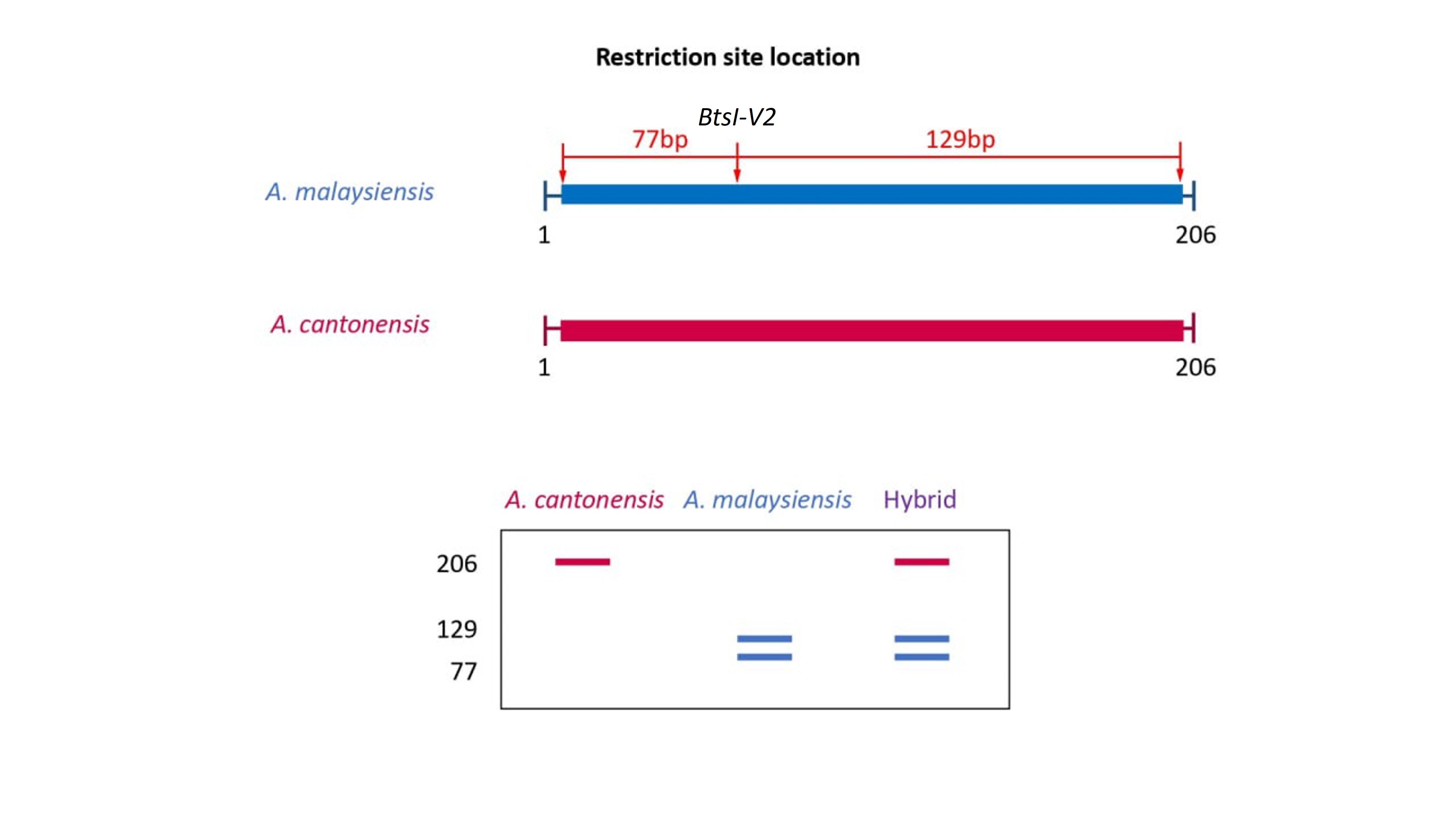

Supplement: Supplementary file 1 — Additional file 1: Figure S1. Illustration of the ITS2 PCR–RFLP band patterns for A. cantonensis, A. malaysiensis, and their hybrid form [file 13071_2024_6140_MOESM1_ESM.jpg]

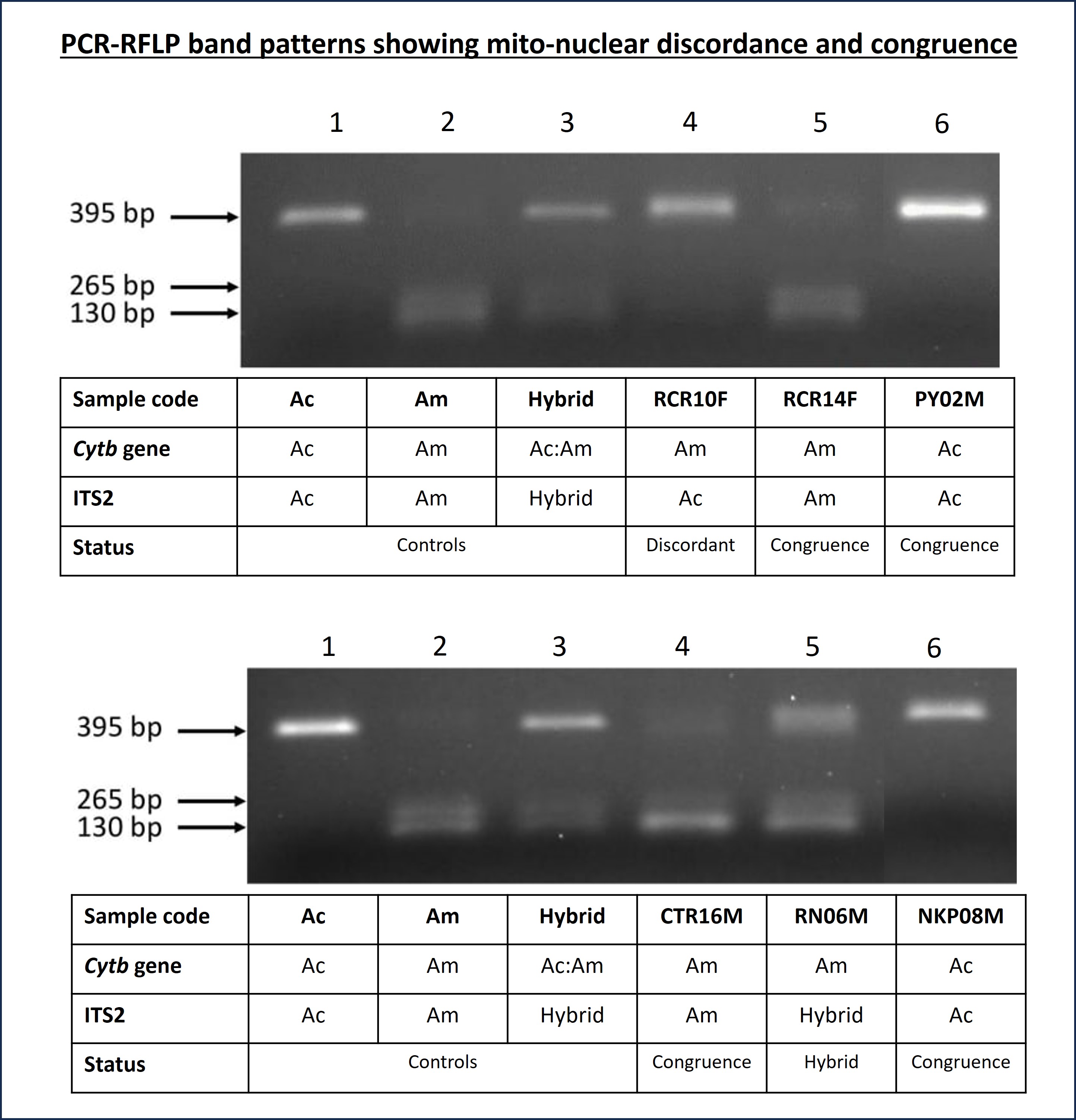

Supplement: Supplementary file 3 — Additional file 3: Figure S2. Examples of ITS2 PCR–RFLP band patterns for specimens showing potential hybrid forms, congruence between the genetic markers, and discordant hybridization [file 13071_2024_6140_MOESM3_ESM.jpg]

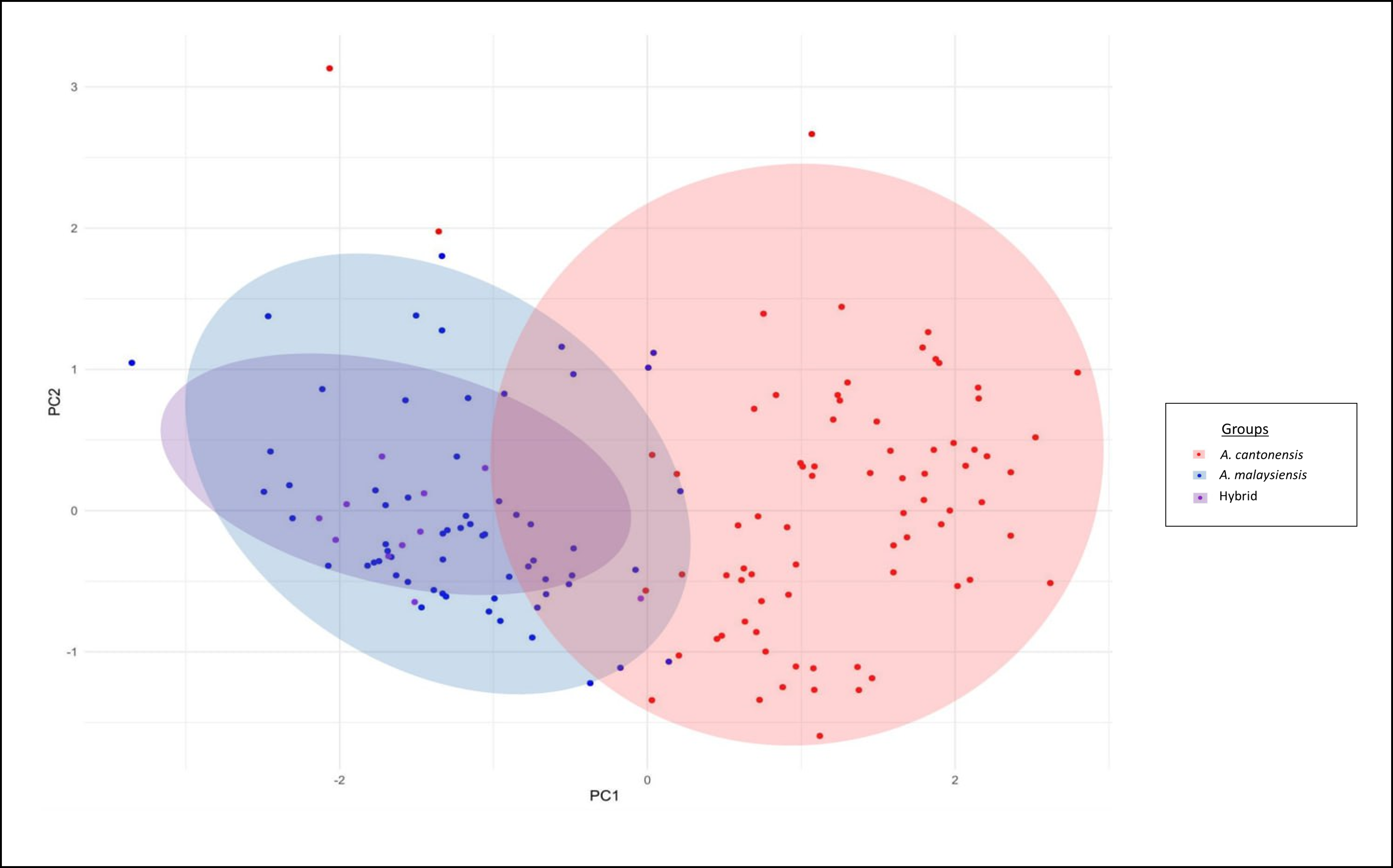

Supplement: Supplementary file 5 — Additional file 5: Figure S3. PCA of male morphological traits for A. cantonensis, A, malaysiensis, and their hybrid form [file 13071_2024_6140_MOESM5_ESM.tif]

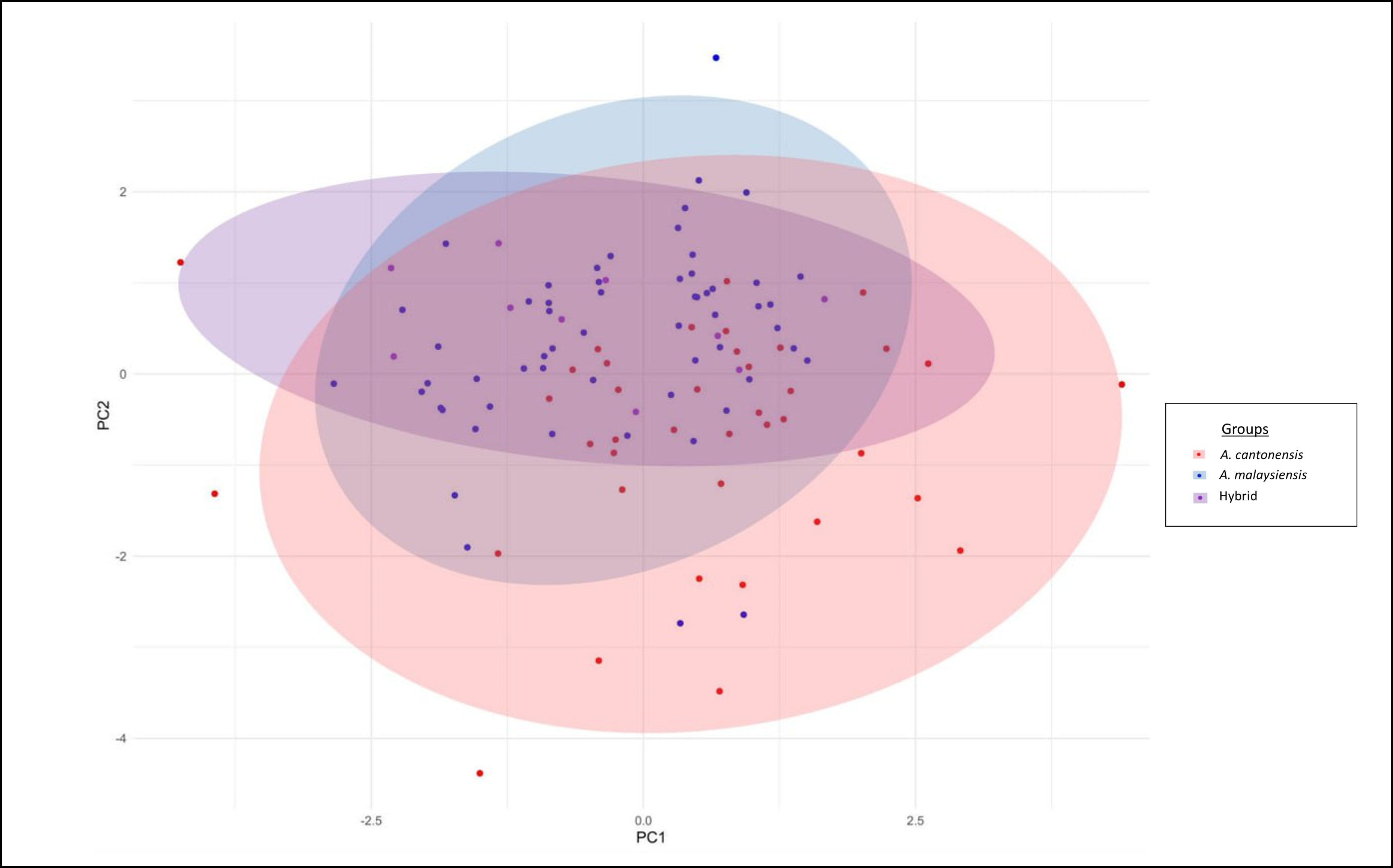

Supplement: Supplementary file 6 — Additional file 6: Figure S4. PCA of female morphological traits for A. cantonensis, A. malaysiensis, and their hybrid form [file 13071_2024_6140_MOESM6_ESM.tif]
